# Unveiling the Aromas and Sensory Evaluation of Hakko Sobacha: A New Functional Non-Dairy Probiotic Fermented Drink

**DOI:** 10.3390/molecules28166084

**Published:** 2023-08-16

**Authors:** Sarah Suffys, Dorothée Goffin, Gaëtan Richard, Adrien Francis, Eric Haubruge, Marie-Laure Fauconnier

**Affiliations:** 1 Laboratory of Gastronomic Sciences, Gembloux Agro-Bio Tech, Liege University, 5030 Gembloux, Belgium; 2 Laboratory of Chemistry of Natural Molecules, Gembloux Agro-Bio Tech, Liege University, 5030 Gembloux, Belgium

**Keywords:** Sobacha, buckwheat tea, functional drink, kombucha, mixed fermentation, volatile organic compounds, stir-bar sorptive extraction GC–MS, OAV, flavor, sensory analysis

## Abstract

At the dawn of a food transition encouraging the consumption of healthy and sustainable non-dairy probiotic products, the development of a fermented functional drink based on Sobacha is considered. Sobacha is an infusion of roasted buckwheat seeds widely consumed in Asian countries for its health benefits. As fermentation improves the nutritional and organoleptic status of grains, the mixed fermentation process involved in the development of kombucha beverages (fermented sweet tea) is conducted by inoculating a symbiotic culture of bacteria and yeasts into the transposable matrix (Sobacha instead of tea). Sobacha, a healthy pseudo-cereal matrix with promising aromas, could be fermented to potentially develop an innovative drink, named “Hakko Sobacha”. This neologism would reveal the fermented character of the infusion, Hakko meaning fermented in Japanese. Considering the beverage characterization, the kinetics of the volatile organic compound syntheses were determined using stir-bar sorptive extraction followed by gas chromatography coupled to mass spectrometry analysis. Odor-active compounds were theoretically calculated to estimate the flavor composition. Finally, sensory analyses highlighted the appreciation and preferences of the consumer towards the beverages. The fermentative yield differences observed between the two buckwheat concentration modalities tested seemed to be correlated with the sugar and nutrient levels available from the starch (buckwheat) matrix. Having characterized Hakko Sobacha, this study proposed the possibility of developing new beverages by monitoring the fermentative process. This should enable improved control and enhancement of their sensorial properties, which could in turn lead to greater customer acceptability.

## 1. Introduction

There is a growing demand for the development of functional foods in the market, i.e., satisfying hunger and nutrient intake, but also preventing chronic diseases and improving physical and mental health. An example of a widely consumed food product is probiotics (provision of beneficial microorganisms (MOs) in the gut microbiota) [1,2]. Thus, in addition to stimulating the growth of beneficial microflora, they have direct effects on both satiety and the intestinal barrier [3]. Moreover, the probiotic market is growing rapidly, opening new opportunities for non-dairy substrates. Thus, pseudo-cereal substrates are promising candidates for the growth of lactobacilli and bifidobacterial strains [4]. Kombucha (from the Japanese “kombu”, algae and “cha”, tea) has gained immense popularity in recent years due to its many health benefits [5]. This fermented drink presents the characteristics of a functional food [6]. Originating in China, Korea, and Japan (220 BC), it is popular for its detoxifying, antioxidant, and energizing properties, as well as against digestive disorders [2,7]. This refreshing beverage is produced by the fermentation of sweet tea, caused by the activity of an MO consortium [8]. The latter is a symbiotic culture of bacteria and yeast (SCOBY), which more commonly refers to the cellulosic substance that develops on the surface of kombucha during fermentation [9].

A combination of alcoholic, acetic, and lactic fermentations occur, as several yeasts and bacteria co-exist in the medium, and the product of one is the substrate for the other [10]. Since ethanol is not only produced during fermentation but also consumed, kombucha is classified as a non-alcoholic beverage because the alcohol content is below 1.2%, considering the European legislation (1169/2011) (0.5% for non-EU countries [11]) characterizing non-alcoholic drinks [12].

Regarding the SCOBY microbial composition [13], *Acetobacter* species generally dominate [14,15], while the genera *Gluconacetobacter* and *Lactobacillus* have occasionally been isolated [16]. Amongst the acetic acid bacteria (AAB), *Acetobacter xylinum* (reclassified as *Gluconacetobacter xylinus* [17]) is considered as the most efficient kombucha cellulose producer [18]. Numerous species of yeast have also been identified, mainly *Zygosaccharomyces* spp., *Saccharomyces* spp., *Dekkera* spp., and *Pichia* spp. [19,20,21].

The aromatic compounds in kombucha are classified into three categories according to their origin: native from the tea substrate, from the saccharide source, and from the metabolites produced by the MO during fermentation [22]. Although kombucha presents a complex mixture of VOCs, only a portion of them contribute to the overall aroma and influence the consumer perception [23].

Finally, this fermented tea with a low sugar content seems to be a healthy alternative to industrial sodas. However, the safety aspects of the drink must also be considered when qualifying the drink as a functional food [24]. Buckwheat tea, also called Sobacha (from Japanese “soba”, buckwheat and “cha”, tea), is a widely consumed infusion in Japan [25]. In Korea, buckwheat tea is known as memil-cha and kuqiao-cha in China [26]. Nowadays, buckwheat is known to be used for its nutritional properties, having established itself as a functional raw material and food additive. More precisely, two species of buckwheat have been commonly cultivated in the world for centuries: common buckwheat (*Fagopyrum esculentum* Moench 1794) and Tartarian buckwheat (*Fagopyrum tataricum* L. Gaertn 1790) [22,27].

In addition, buckwheat has been used as an effective medicinal plant in traditional Chinese therapy in the treatment of many diseases for over 1000 years [28]. It has been shown to lower plasma cholesterol levels, provide neuroprotection, have anti-cancer, anti-inflammatory and anti-diabetic effects, and reduce hypertension [26]. Buckwheat, described as a pseudo-cereal [29], is an interesting food matrix for celiacs (people suffering from gluten sensitivity or intolerance) [30]. In this way, as Sobacha is a popular health product in Asian and European countries [31,32,33], considering how the processing of buckwheat into tea affects the bioactive compounds in the grain is of great interest [26]. The processes and know-how for making Sobacha are patented and mostly originate from China and Japan [34,35,36]. It is in these countries that one finds the darkest infusions, a sign of their richness in nutrients and aromas. Globally, before being removed from the hull, the raw whole seeds are soaked in water, steamed, and dried. Then, the hulled groats are roasted to obtain the kasha (roasted buckwheat seeds) that will be in turn used to make the infusion.

The physico-chemical process consists of hydrolyzing the intermolecular bonds of buckwheat starch to allow the hydrogen bonding sites to bind to water molecules. Thus, the nutrients and simple sugars (mainly glucose units) from the buckwheat seed are made available in the infusion, as the accessibility of the molecules composing the matrix allows its aromatic development.

Sobacha is mainly prepared with kasha, but the literature also refers to the use of roasted leaves or flowers from the plant. However, the seeds are still the main source of nutrients and flavors, which are then found in the infusion. The addition of teas to Sobacha during brewing is a common practice, mostly implemented for taste improvement (addition of tea flavors).

Drinking buckwheat infusion has many health benefits, including improving heart health, preventing kidney complications, reducing the risk of cancer, and encouraging weight loss. The soluble fibers (mainly pectin and other polysaccharides) released during the infusion help to normalize blood sugar, insulin, and cholesterol levels. The insoluble fibers help to ensure optimal digestive transit. The content of minerals such as magnesium helps to reduce oxidative stress. Sobacha contains few calories and is therefore a substitute for teas. In addition, it can be consumed without requirements as it does not contain any stimulating active molecule on the consumer metabolism (as compared to theine present in tea) and can have a beneficial effect on patients suffering from chronic venous insufficiency, preventing the formation of edema. In addition to its health benefits, the infusion is appreciated for its flavor as the roasted seeds bring a light and distinctive aroma of praline and hazelnut. Cereals and pseudo-cereals are considered one of the most important sources of dietary protein, carbohydrates, vitamins, minerals, and fiber in the world. However, the nutritional quality and the sensory properties of their products are sometimes inferior to those of animal products such as milk and dairy products. The main causes are lower protein content, deficiency or low levels of certain essential amino acids, particularly lysine, and the presence of anti-nutritional factors such as phytic acid, tannins, and polyphenols. On the other hand, the nature of grains is an alternative to animal-based dairy products.

In this context, fermentation can improve the nutritional status of pseudo-cereal products. In general, their fermentation leads to a decrease in carbohydrates and certain non-digestible poly- and oligosaccharides. Some amino acids can be synthesized, and the availability of B vitamins can be improved.

Fermentation also provides optimal conditions regarding the optimal enzymatic degradation of phytates, which are present in pseudo-cereals in the form of complexes with polyvalent cations such as iron, zinc, calcium, magnesium, and proteins. Indeed, fermentation leads to an overall improvement in the shelf life, texture, taste, and aroma of the final product. Thus, functional fermented beverages based on pseudo-cereals are becoming increasingly attractive as healthy alternatives for consumers with lactose and gluten intolerances. Sobacha, a healthy pseudo-cereal matrix with promising aromas, could be fermented to potentially develop innovative flavors. In addition, the neologism *Hakko Sobacha* would reveal the fermented character of the infusion, *Hakko* meaning fermented in Japanese.

The research questions that this manuscript attempts to answer are the following:

- Does Sobacha fermentation induce the acidification of the medium towards a pH range allowing the inhibition of pathogenic microorganisms?
- What are the production kinetics of the molecules of interest in the degradation of fermentable sugars? Are the levels of ethanol produced at the end of fermentation in line with the standards for designating non-alcoholic beverages?
- What are the major volatile organic compounds developed during Sobacha fermentation? Among these, which aromas contribute significantly to the product’s final aromatic profile?
- From a sensory point of view, what are the consumer’s opinions and preferences regarding the beverages formulated?

To address these issues, after analyzing the physico-chemical parameters of the formulated beverage as a function of the fermentation key parameters (such as the fermentation time), the kinetics of the volatile organic compound syntheses were determined using stir-bar sorptive extraction followed by analysis with gas chromatography coupled to mass spectrometry (SBSE–GC–MS); SBSE allows for then optimal capture of volatile and semi-volatile compounds in aqueous matrices. The correlation with microbial metabolic pathways was then established, ensuring a constant quality and stability of the produced metabolites regarding the Sobacha substrate.

Although kombucha presents a complex mixture of VOCs, only a portion of them contribute to the overall aroma and influence consumer perception. Ortho-nasal detection odor thresholds were then considered to determine odor-active compounds and evaluate theoretical sensory profiles of Hakko Sobacha. Finally, the aromatic compound kinetics during the fermentation process were coupled with sensory analysis. This methodology will in time allow for the design of new drinks by controlling the fermentation process, as well as meet consumer preferences.

## 2. Results and Discussion

### 2.1. Physico-Chemical Characterization of Hakko Sobacha during Fermentation

#### 2.1.1. Evolution of pH during Fermentation

**Figure 1:**
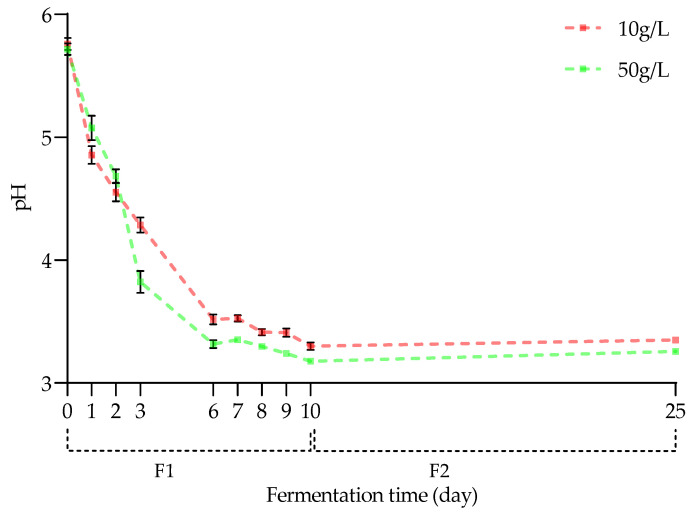
pH evolution of Hakko Sobacha samples as a function of kasha concentration (10 and 50 g/L) and fermentation time (day) at 25 °C (*n* = 3).

As illustrated in Figure 1, the pH values of Hakko Sobacha exponentially decreased during the first aerobic fermentation. The pH was revealed to be non-significantly different between the first and second fermentation days for both kasha concentrations considered (means of 5.76 ± 0.05 and 5.71 ± 0.05 g/L at D0 to 4.55 ± 0.07 to 4.68 ± 0.05 g/L at D2, for 10 and 50 g/L kasha concentration modalities, respectively). From the third day (D3) until the end of F1, the pH decrease started to be significantly different between the two kasha concentration modalities and fermentation times, with higher pH values for the fermentation medium with the lower kasha concentration (means of 4.29 ± 0.06 and 3.82 ± 0.09 g/L (*p* < 0.01) at D3 to 3.30 ± 0.03 and 3.18 ± 0.01 g/L (*p* < 0.01) at D10, for 10 and 50 g/L kasha concentration modalities, respectively). A significant increase in pH occurred during the second anaerobic fermentation, reaching mean values of 3.35 ± 0.02 and 3.26 ± 0.01 g/L (*p* < 0.01) at D25 (for 10 and 50 g/L kasha concentration modalities, respectively).

When comparing Hakko Sobacha and kombucha pH evolution trends, similar behaviors should be observed, namely an exponential decrease followed by a slower decrease, as often mentioned in the literature for the kombucha fermentation process. Moreover, studies focusing on the anaerobic stage of the kombucha fermentation process also observed a slight pH increase over time. However, given the lower polyphenol content in the buckwheat infusion compared to the tea matrix, the significant difference in pH observed at the initial time could be linked to the lower acidity of the infusions (initial pH of 4.05 for a black/green tea kombucha).

Therefore, the pH decrease may reflect the acidification of both fermentation media and thus the microorganism’s ability to metabolize diverse products in terms of Sobacha composition. Since this acidification is accentuated when the kasha concentration is higher, the hypothesis that the presence of an environment richer in carbon sources and nutrients available for metabolic activity, such as in buckwheat, could be advanced.

#### 2.1.2. Evolution of SCOBY Production during Fermentation

**Figure 2:**
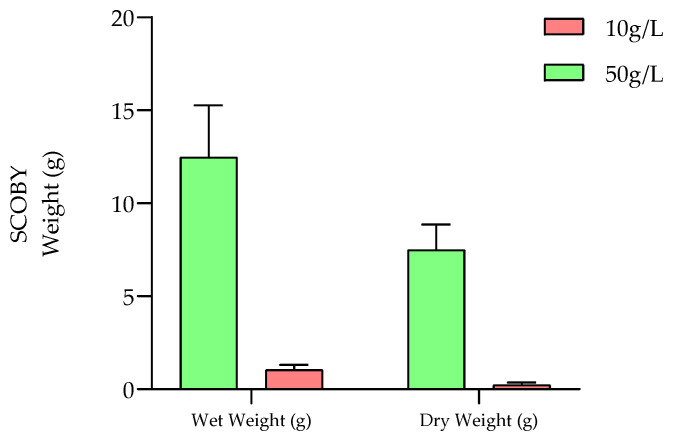
SCOBY weights (g) at final F1 fermentation stages (D10) as a function of the kasha concentration (10 and 50 g/L) at 25 °C (*n* = 3).

Microbial cellulose growth is a direct parameter for assessing the microorganism’s evolution in the fermentation medium. More precisely, it is directly linked to the ability of most *Acetobacter* and *Gluconobacter* genera to synthetize cellulose from available carbon sources. Subsequently, the evolution of SCOBY weights as a function of kasha concentration is presented in Figure 2.

From an initial SCOBY inoculum of ±5 g for both concentration modalities, the subsequent nascent SCOBY pellicle weights increased significantly during the aerobic fermentation process. Indeed, significant differences were observed for the resulting cellulose growth as a function of the buckwheat concentration (wet weight means of 1.05 ± 0.26 and 12.47 ± 2.80 g (*p* < 0.01) for 10 and 50 g/L kasha concentration modalities, respectively).

Concerning dry matter, very high significant differences were reported (dry weight means of 0.23 ± 0.13 and 7.49 ± 1.37 g (*p* < 0.001) for 10 and 50 g/L kasha concentration modalities, respectively). This could be related to the absorption capacity of the cellulose films generated (closely linked to the thickness of the biofilms), allowing liquid to be retained to a greater or lesser extent.

These observations are in accordance with the different acidification profiles obtained from kasha concentration modalities (Section 2.1.1.) and the carbon sources available for cellulose production during the aerobic fermentation step. Indeed, the presence of a higher simple sugar content when the buckwheat concentration is increased allows for both the microorganism’s metabolic activity, which is responsible for cellulose synthesis, and acidification to be improved.

#### 2.1.3. Carbohydrate, Alcohol, and Organic Acid Concentration Kinetics during Hakko Sobacha Fermentation

**Figure 3:**
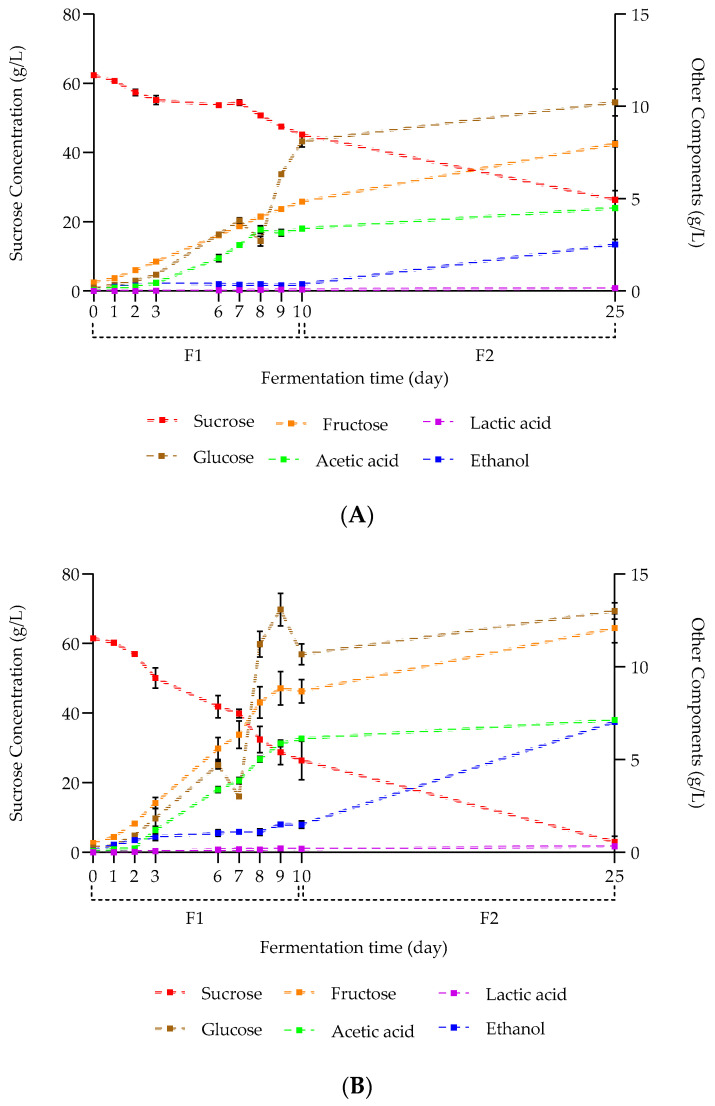
Evolution of the concentration of carbohydrates, alcohol, and organic acids (g/L) in Hakko Sobacha as a function of kasha concentration (10 g/L (**A**) and 50 g/L (**B**)) and fermentation time (day) at 25 °C (*n* = 3).

The evolution of carbohydrates, organic acids, and ethanol contents as a function of buckwheat concentration and fermentation time is illustrated in Figure 3.

Considering the carbohydrate analysis, the initial sucrose contents decreased over time. From the initial stage to D3, no significant difference was observed regarding the kasha concentration (means of 62.36 ± 0.38 and 61.50 ± 0.50 g/L at D0, 55.19 ± 0.15 and 50.08 ± 0.63 g/L at D3, for 10 and 50 g/L kasha concentration modalities, respectively). From D6, the sucrose contents showed significant differences from the kasha concentrations (means of 53.69 ± 0.15 and 41.87 ± 0.32 g/L (*p* < 0.01) at D6, to end with 26.37 ± 2.65 and 3.02 ± 1.62 g/L (*p* < 0.001) at D25, for 10 and 50 g/L kasha concentration modalities, respectively). On the other hand, the amounts of residual sucrose reported in the literature for kombucha vary according to the initial sucrose input.

The buckwheat concentration therefore seemed to have a great impact on the metabolic activity of sucrose conversion by the microorganisms during the first fermentation. In addition, the significant difference in sugar levels observed at the end of F2 as a function of buckwheat concentration could be explained by the modification of the fermentation medium. Indeed, the microbial consortium could benefit from a larger amount of nutrients present in the infusion and subsequently released during fermentation. In other words, the starch and complex buckwheat sugars released in the infusion and partially hydrolyzed by the microbiome represent another source of carbohydrates, enabling them to further acidify the medium.

The degradation kinetics of sucrose into glucose and fructose units by yeast taxa via the glycolysis pathway ensures that simple carbohydrates are assimilated by the latter. Consequently, as the simple sugar level is inversely proportional to that of sucrose, it increases during fermentation [2].

Indeed, the glucose and fructose contents increased significantly over time, with a higher concentration of fructose at all points (except for the second fermentation). Moreover, the medium exhibited higher levels of simple sugars when the buckwheat concentration in the fermentation medium was higher. The initial glucose levels were 0.19 ± 0.02 and 0.26 ± 0.03 g/L (*p* < 0.05), and the final levels reached 10.22 ± 0.73 and 13.00 ± 0.44 g/L (*p* < 0.01) for the 10 and 50 g/L kasha concentration modalities respectively.

During the hydrolysis of sucrose, yeast concurrently converts both fructose and glucose into ethanol and CO_2_ on one side, with a more rapid utilization of glucose compared to fructose. Additionally, the preferential carbon source for AAB is glucose, leading to the synthesis of microbial cellulose. Since Sobacha is a food matrix that is rich in starch and made up of glucose units, the main source of fructose in the fermentation medium is sucrose.

The ethanol levels significantly increased as a function of kasha concentration during the fermentation process. Starting from 0.04 ± 0.01% and 0.10 ± 0.01% (*p* < 0.01) at D0 to 0.05 ± 0.02% and 0.19 ± 0.02% (*p* < 0.01) at D10 during the aerobic step, ending with ethanol levels of 0.32 ± 0.03% and 0.89 ± 0.01% (*p* < 0,0001) at D25 for the anaerobic process (for 10 and 50 g/L kasha concentration modalities, respectively). Ethanol synthesis at the beginning of fermentation is indeed reported in the literature and correlated with an exponential increase in the yeast population. Moreover, studies reported a kombucha final ethanol content of approximately 0.5% and 0.6% after 12 and 20 days of fermentation, respectively (for initial sucrose contents of 50 and 100 g/L, respectively), which is in between the results for Sobacha.

Yeasts drive ethanol production thanks to the availability of carbonaceous and nitrogenous substrates as well as oxygen in the medium. A further increase in ethanol occurs during the anaerobic fermentation step, as yeasts become the dominant microorganism in the medium, to the detriment of bacteria. The availability of additional glucose from starch hydrolysis during the anaerobic step allows for obtaining a higher final ethanol content.

At the end of the anaerobic fermentation, both beverage’s ethanol levels were below the 1.2% (*v*/*v*) considered by the European regulation (1169/2011) for characterizing non-alcoholic drinks.

Furthermore, due to the presence of a symbiotic ecosystem in Hakko Sobacha, the ethanol produced by the yeasts was then converted via multiple metabolic pathways into organic acids by different bacteria genera. In this way, the acetic acid contents increased progressively over time, characterized as significantly different from D3 as a function of kasha concentration. The acetic acid levels started from 0.06 ± 0.01 and 0.07 ± 0.01 g/L (*p* > 0.05) at D0 until 4.49 ± 0.13 and 7.13 ± 0.12 g/L (*p* < 0.001) at D25 (for 10 and 50 g/L kasha concentration modalities, respectively).

Previous studies focused on traditional kombucha reported a significant initiation of acetic acid production at D3 (reaching approximatively 5 g/L for an initial sucrose level of 100 g/L) and an exponential increase in bacteria population growth from this stage.

Despite the low concentrations of lactic acid measured during the fermentation process, they increased significantly over time starting from D3, depending on the kasha concentration. The lactic acid production started at 0.02 ± 0.01 and 0.07 ± 0.01 g/L (*p* < 0.01) at D3 to reach final values of 0.15 ± 0.03 and 0.33 ± 0.03 g/L (*p* < 0.01) at D25 (for 10 and 50 g/L kasha concentration modalities, respectively). Indeed, LAB represent a minor part of the microbial consortium present in the kombucha SCOBY compared to AAB; lactic acid is then less produced.

### 2.2. Study of the Volatile Organic Compound Development Kinetics during Fermentation

#### 2.2.1. Chemical Analysis of Major VOCs

The evolution of Hakko Sobacha VOC levels as a function of fermentation time and buckwheat concentration are presented in Table 1 and Table 2 (for kasha concentration modalities of 10 and 50 g/L, respectively).

A total of 43 VOCs were reported in Hakko Sobacha (10 g/L kasha level modality) during the fermentation process, comprising 5 alcohols, 5 aldehydes, 12 carboxylic acids, 9 esters, 4 ketones, 2 phenols, 3 pyrazines, and 3 terpenes (Table 1). The VOC profile obtained with a more concentrated buckwheat beverage (Table 2, 50 g/L modality) during fermentation exhibited 54 components, namely 4 alcohols, 4 aldehydes, 1 benzothiazole, 10 carboxylic acids, 11 esters, 5 ketones, 4 phenols, 10 pyrazines, 1 pyrrole, and 4 terpenes. In fact, the most concentrated infusion allowed for the enrichment of the diversity of molecules developed during fermentation.

The Sobacha matrix before fermentation—considering all kasha concentration modalities—was mainly composed of carboxylic acids, terpenes, and pyrazines.

The buckwheat infusion presented high levels of palmitic (650.24 and 304.18 ppm) and palmitoleic acids (180.24 and 144.70 ppm, for 10 and 50 g/L kasha concentration modalities, respectively). In fact, fatty acids are a specific type of carboxylic acids in tea and infusion matrices. Another major class from plant-based infusions are terpenes, presenting properties involved in the plant chemical defense. The major Sobacha terpenes were p-cymene (676.32 and 510.21 ppm) and m-myrcene (510.21 and 439.92 ppm, for 10 and 50 g/L kasha concentration modalities, respectively). Lastly, pyrazines present in the infusion are for the most part related to the buckwheat seed roasting process. Thus, pyrazine levels are positively correlated with kasha concentration. The major pyrazine representatives present in the Sobacha were 2-methoxy-5-methylpyrazine (342.21 ppm) and 2-ethylpyrazine (129.11 ppm) for the 50 g/L kasha concentration modality.

**Figure 4:**
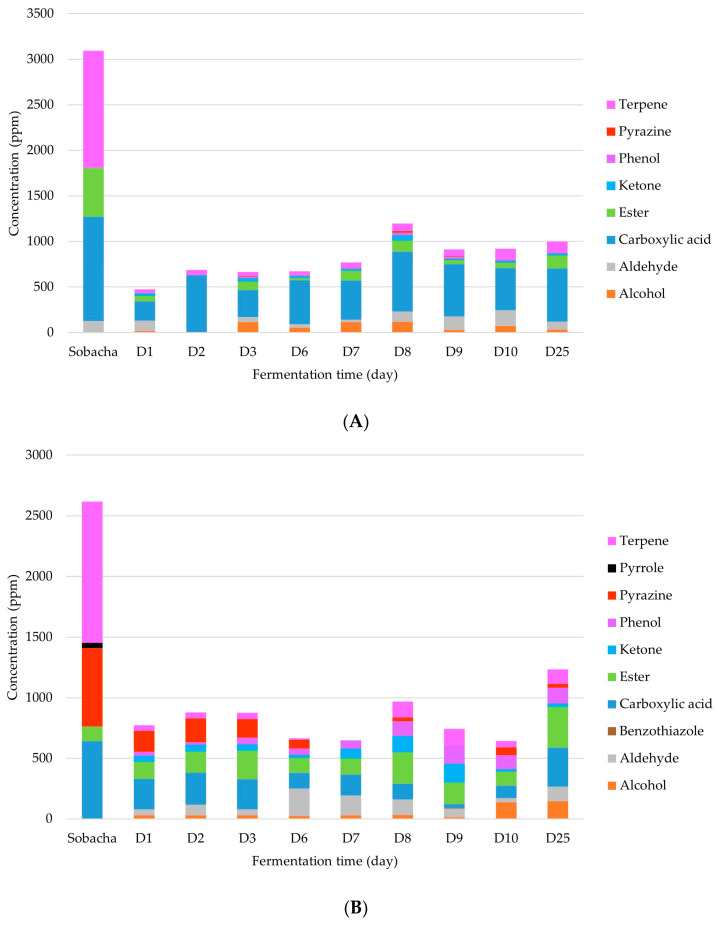
Changes in average contents (ppm) of different classes of VOCs with Hakko Sobacha (kasha concentration of 10 g/L (**A**) and 50 g/L (**B**)) fermentation time (day) at 25 °C.

Figure 4 graphically illustrates the average content changes of all compound classes as a function of buckwheat concentration and fermentation time, and a link between the VOC diversity profile and the total content of released compounds could be established during the fermentation process. In fact, fermentation dynamically changes the VOC profile of the starting infusion into a different mixture of compounds.

The classes present in the initial infusion tended to decrease during fermentation. As fermentation started, the microbial consortium activity assimilated the organic substrates present in the infusion media to synthetize metabolites, consequently leading to new VOC diversity and proportions.

As described by Suffys et al. (2023) [23], classes such as alcohols, ketones, aldehydes, and esters are the main metabolites synthesized by the microbiome in kombucha-type fermentation. Indeed, the related metabolic pathways could be similar and adapted to the nutrients present in the infusion.

Carboxylic acids are also major components produced during the fermentation process, followed by esters. The final average contents reported for carboxylic acids were 587.30 and 322.45 ppm, and for alcohols 30.91 and 146.94 ppm (for 10 and 50 g/L kasha concentration modalities, respectively). Indeed, it has been reported in previous studies on kombucha that these two classes of molecules constitute substrates for the synthesis of other types of compounds such as esters, phenols, and terpenes mainly (molecules formed using aldehydes and ketones).

Moreover, mixed fermentation allows to produce enriched VOC profiles by non-Saccharomyces yeast strains such as *Zygosaccharomyces* sp., *Dekkera* sp., *Candida* sp., and *Pichia* sp. They contribute to the final product aroma but also to the formation of organic acids by their oxidation. Principally, together with aldehydes, alcohols are the main substrates used by the MO present in the SCOBY to synthesize aromatic volatile acids that acidify the medium and esters.

Intermediary classes such as aldehydes and ketones are converted during fermentation into secondary metabolites and aromatic molecules. Indeed, their contents at the end of the fermentation process were relatively low (86.97 and 119.73 ppm for aldehydes, 15.64 and 28.16 ppm for ketones, for 10 and 50 g/L kasha concentration modalities, respectively).

Finally, focusing again on the VOC classes mainly formed during the fermentation process, the total ester contents reached 143.70 and 335.34 ppm, and for phenol 24.38 and 128.56 ppm, for the 10 and 50 g/L kasha concentrations, respectively. The major ester synthetized was 2-phenylethyl acetate (final contents of 120.67 ± 8.41 and 57.55 ± 2.20 ppm, for 10 and 50 g/L kasha level modalities, respectively). Developed by the acetic acid condensation reaction, this ester participates in kombucha-type fermentation. In this way, ethyl caprylate (95.52 ± 1.29 ppm at D25, for 50 g/L of kasha) was the major ester metabolized from the ethanol substrate. At last, 4-ethylguaiacol was the major phenol found in Hakko Sobacha (116.18 ± 3.33 ppm at D25, for 50 g/L of kasha), an aromatic phenol produced by the yeast genus *Brettanomyces*.

**Figure 5:**
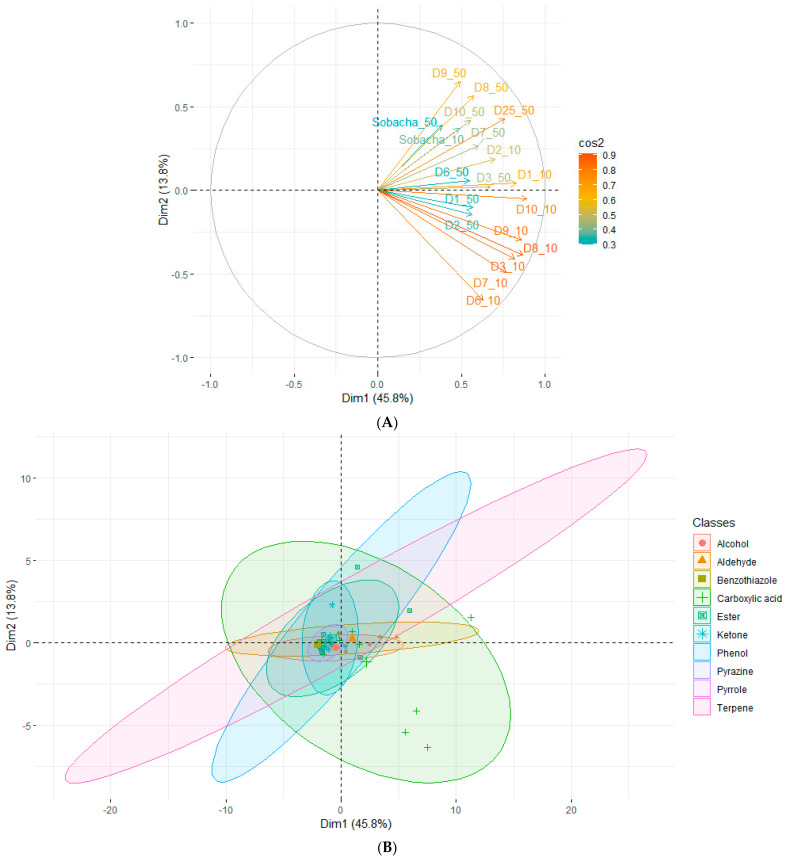
Principal component analysis (mean values for VOC averaged across Hakko Sobacha fermentation time (day) and kasha concentration (_10 for 10 g/L and _50 for 50 g/L)). Plots of variable correlations (cos^2^ color scale) (**A**) and individual representations (ellipses correspond to VOC classes) (**B**). Labeled individuals are those best represented on the plan.

A PCA was performed to analyze the variation among the different VOC profiles during the fermentation process (Figure 5). The first dimension explained 45.9% of the total variance, represented on the horizontal axis. A variable correlation plot illustrates the possible group formations, depending on their position on the 1:2 plan (Figure 5A). First, the infusions—considering all kasha concentrations—were positively correlated together, inducing similar VOC profiles. The overall fermentation process differed according to the kasha concentration in the infusion, as the variables were situated in fourth quadrant (QIV) and first quadrant (QI) (for 10 and 50 g/L kasha, respectively). Moreover, the contribution scale indicated that the data set variability was further explained by the Hakko Sobacha beverage based on the 10 g/L kasha concentration modality. Finally, considering all kasha concentrations, the fermentation stage groups traducing similar VOC profiles were the following: D1–D2, D3–D7 and D8–D25. Moreover, the trends provided by the second dimension revealed no additional information considering this dataset (explanation of 13.7% of the total variability). To resume, a major significant change in the VOC profiles between the beginning (D1–D2), the intermediate (D3–D7), and the end (D8–D25) of the fermentation stages was highlighted by the PCA. Furthermore, as illustrated by the individual representations (Figure 5B), the classes that explained the major variability between kasha concentrations and fermentation times were carboxylic acids, terpenes, aldehydes, and phenols (most represented along Dim1).

#### 2.2.2. Theoretical Aromatic Characterization of Hakko Sobacha and Estimation of Sensory Profiles

As the microbial consortium plays a crucial role in the development of flavor quality during fermentation, the formulated beverages will now be analyzed from this point of view.

The evolution of aromatic molecules is presented in Table 3 and Table 4 (for 10 and 50 g/L kasha concentration modalities, respectively). Based on the VOC levels and perception thresholds in water reported in the literature, odor-activity values were determined during the fermentation process. The odor-activity value (OAV) explains the contribution of a VOC to a specific aroma. When the OAV is greater than 1, it indicates that the considered compound has a significant contribution to the overall aroma in the corresponding food system. Indeed, the greater the OAV is, the higher the contribution of the VOC to the aroma is.

**Figure 6:**
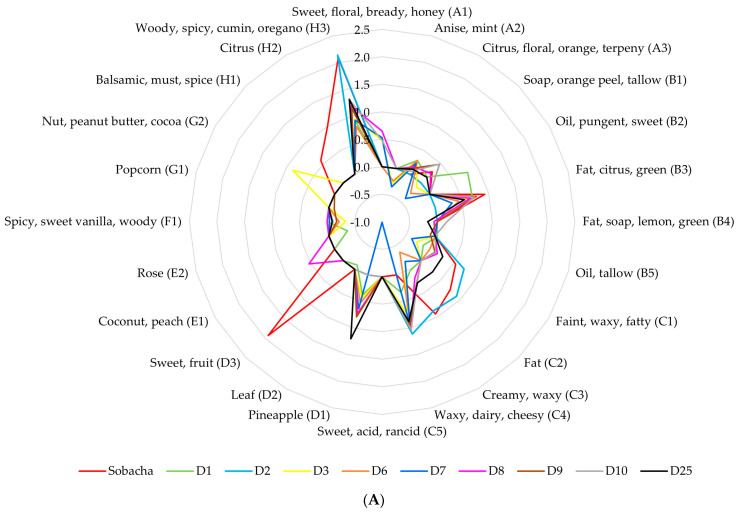

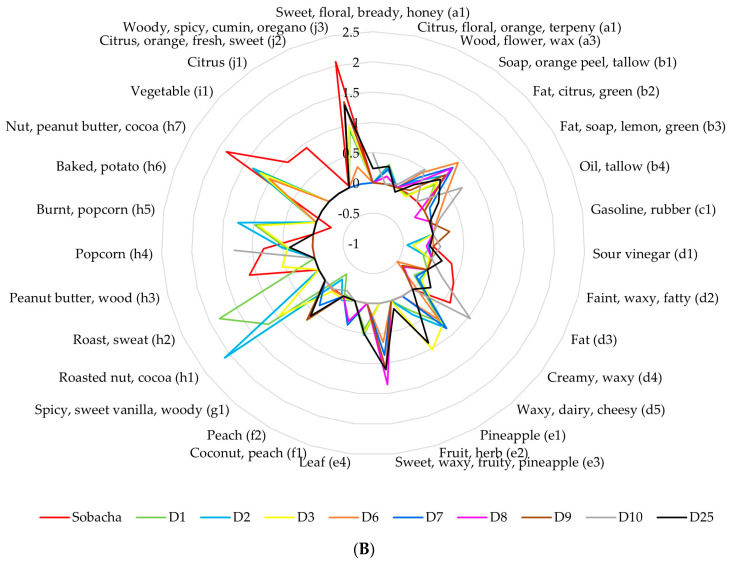
Estimated aromatic profiles expressed as the log of the OAV for VOCs (% area > 5%) with Hakko Sobacha ((**A**) kasha concentration of 10 g/L and (**B**) of 50 g/L) fermentation time (day) at 25 °C (OAV = concentration/perception threshold).

The analysis of the perceived aromatic compounds in the less concentrated buckwheat beverage (10 g/L, Table 3) highlighted 24 VOCs: 3 alcohols (A), 5 aldehydes (B), 5 carboxylic acids (C), 3 esters (D), 2 ketones (E), 1 phenol (F), 2 pyrazines (G), and 3 terpenes (H). On the other hand, when analyzing the higher concentrated beverage (50 g/L, Table 4), an increase in the diversity of the perceived molecules was observed, as it comprised 31 VOCs: 3 alcohols (a), 4 aldehydes (b), 1 benzothiazole (c), 5 carboxylic acids (d), 4 esters (e), 2 ketones (f), 1 phenol (g), 7 pyrazines (h), 1 pyrrole (i), and 3 terpenes (j). The introduction of a code including their class for each perceived molecule (letter followed by a number) in Table 3 allows for their clear identification in the sensory profiles (Figure 6).

The most characteristic aromatic molecules of the low-buckwheat infusion were nonanal (B3) (OAV of 8.49), palmitic acid (C3) (OAV of 8.67), linalyl acetate (D3) (85.15) and the terpenes m-myrcene (H2) (9.72) and p-cymene (H3) (122.97). However, despite their similar profiles regarding terpenes (m-myrcene OAV of 8.38 and p-cymene OAV of 122.97), the most representative active molecules of the more concentrated buckwheat infusion were the pyrazines 2-ethylpyrazine (h3) (OAV of 12.91) and 3,5-diethyl-2-methylpyrazine (h7) (OAV of 72.24). In terms of flavors, the light-buckwheat infusion presented fat–citrus–green, creamy–waxy, sweet–fruit, and woody–spicy–cumin–oregano aromas. On the contrary, typical kasha aromas were found in the more concentrated matrix, such as peanut butter–wood–nut and cocoa. The latter also presented major terpene notes: citrus and woody–spicy–cumin–oregano.

During the fermentation process, the Sobacha sensory profile was modified through MO activity, resulting in the development of diverse aromatic molecules with different perception profiles. The synthesis of 2-phenylethyl acetate (final OAV of 16.09 and 7.67 for 10 and 50 g/L kasha concentration modalities, respectively) contributed a pineapple aroma to the final beverage’s flavor. Ethyl caprylate was another ester metabolized during fermentation, bringing sweet–waxy–fruity–pineapple notes (final OAV of 12.74 for the kasha concentration modality of 50 g/L). Pelargonic acid, giving waxy–dairy–cheesy aromas, also contributed to the final Hakko Sobacha flavor (final OAV of 7.51 for the kasha concentration modality of 10 g/L). However, even if the initial infusion constituents globally decreased across the fermentation stages, they still contributed to the aroma system as their OAV remained greater than 1.

The correlation between the changes in VOC profiles during the fermentation process and metabolic pathways is reported in the literature. Therefore, aroma-active compounds also fluctuate. Considering the metabolic pathways employed in kombucha-type fermentation, multiple aromatic compounds are referred to as microbial. However, although the beverage’s final flavor maintains common compounds that contributes to the kombucha-type fermentation aromatic signature, typical aromatic compounds from its original substrate allow for the obtention of a fresh drink with innovative flavors. The Hakko Sobacha final flavor also depends on fermentation parameters such as the initial sucrose concentration and the liquid starter age and concentration, the microbial composition of the SCOBY, temperature, and time.

### 2.3. Study of Sensory Analysis Trends Resulting from the Hakko Sobacha Tasting

Initially, the consumer sample surveyed was predominantly male (57%), mainly in the 18–25 age bracket (54%) (followed by the 26–35 age bracket with 35%), and mainly university students (58%, versus 42% employees). In terms of consumption habits, 88% of consumers were familiar with fermented beverages. In contrast, 37% of the population had never tasted these drinks, followed by 28% who consumed them once a trimester and 18% several times a month. Kombucha was the most widely consumed beverage (87%), followed by kefir (45%). Finally, 75% of the population sampled bought these drinks in stores, and 55% made them at home.

**Figure 7:**
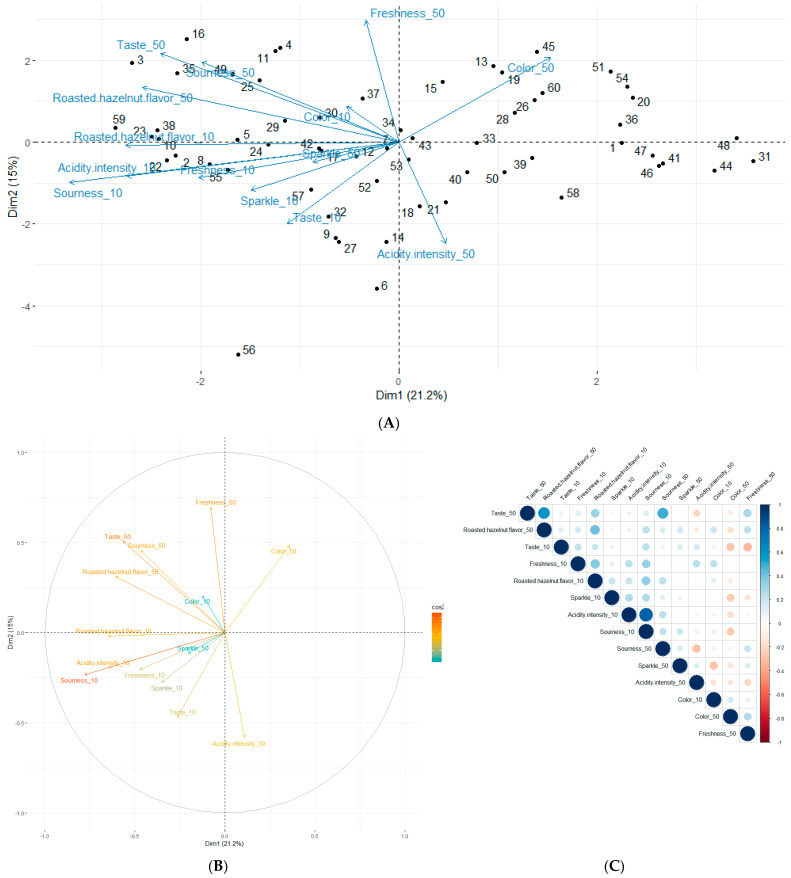
Principal component analysis plots of Hakko Sobacha sensory evaluation variables and individuals (**A**), variable correlations (cos^2^ color scale) (**B**) and correlations between different sensory descriptors regarding the kasha concentration (_10: 10 g/L and _50: 50 g/L) (a color gradient denotes the Spearman’s correlation coefficients) (**C**).

The data resulting from the sensory analysis carried out on the formulated beverages were studied using PCA, as referred in Figure 7. However, since PCA explained only 36.2% of the total dataset variability in the 1:2 dimensions, a complementary analysis resulting from the triangular and hedonic tests was envisaged in order to draw more robust conclusions. The PCA variable dispersions regarding both drinks tested may illustrate the consumer’s ability to perceive them differently. These trends were confirmed by the triangle test results, traducing the population’s ability to differentiate the two drinks offered in the test (*p* < 0.0001). Descriptors concerning the drink with a kasha concentration of 10 g/L seemed to be grouped together in quadrant III, while those describing the 50 g/L kasha concentration were in quadrant II (Figure 7A). In addition, the variable contributions explaining the data variability (Figure 7B) might be the sourness and taste, followed by the roasted hazelnut aroma taste and the freshness of the drinks. The Spearman’s correlation between the descriptors and kasha concentration modalities explained the descriptor contribution, as the quantitative score assessed by each judge regarding all descriptors allowed for the identification of links from one perceived descriptor to another (Figure 7C). Considering the positive correlation between the sensorial descriptors, the most linked attributes were acidity intensity–sourness, then taste–roasted hazelnut aroma, and taste–sourness. These results underlined the fact that the descriptors were correlated for a given beverage and were therefore perceived in different ways from one beverage to another. In this way, negative correlations therefore concerned descriptors for different beverages.

To go deeper into the beverage’s evaluations and judge preferences, the individual clusters grouped by appreciation similarities as a function of biplot quadrants in the first two dimensions was envisaged (Figure 7A). The QI cluster generally disliked the two drinks tasted for their acidity, taste, fizz, and hazelnut flavor. The QIII cluster, mirroring the previous group, seemed to like both drinks. A preference for the drink with the highest buckwheat concentration (50 g/L) was demonstrated by the intensity of the following variables: acidity and intensity, freshness, fizz, taste, and roasted hazelnut aroma. These outlines were confirmed by the pairwise test results, as the population proportion that preferred the highest buckwheat-concentrated drink for the following descriptors: acidity (63.33% (*p* < 0.001)), intensity (53.33% (*p* < 0.01)), fizz (55% (*p* < 0.01)), taste (63.33% (*p* < 0.001)) and roasted hazelnut aroma (73.33% (*p* < 0.0001)). From a global point of view, this beverage preference reached 57% (*p* < 0.001). The two remaining clusters seemed also opposed, where the group of individuals in the QII cluster appreciated both drinks for the characteristic’s acidity, hazelnut aroma, intensity of acidity, taste, and freshness of drink_50 (kasha 50 g/L) on the one hand and taste, acidity, and hazelnut aroma of drink_10 (kasha 10 g/L) on the other. Individuals in the QIV cluster found the color of drink_50 pleasant but the acidity and hazelnut flavor unsatisfactory. Finally, the QIV cluster did not like drink_10 for the following characteristics: acidity, taste, intensity of acidity, hazelnut flavor, freshness, and fizz. The color attribute describing drink_10 (kasha 10 g/L) reached 53.33% (*p* < 0.01). There was no significant difference (*p* > 0.05) observed regarding the freshness descriptor (accounted for 51.67% and 48.33% concerning drink_50 (kasha 50 g/L) and drink_10 (kasha 10 g/L), respectively).

Finally, the hedonic evaluation mentioned that the preferred times for Hakko Sobacha consumption were during the afternoon (67%) and as an aperitif (52%). The preferred quantity (71%) was the equivalent of a water glass (25 cL). The judges’ most frequent comments on the drinks were pleasant (44%), followed by a non-neglectable high acidity and odor (15%). As a result, the major judges surveyed (80%) were ready to incorporate Hakko Sobacha into their daily routine as a more natural substitute for soft drinks.

## 3. Materials and Methods

### 3.1. Materials and Chemical Reagents

Kasha seeds used to prepare Sobacha infusion were sourced from Japan (Shizuoka). The Chun Mee Moon Palace green tea and Lien Son black tea leaves used in the fermentation liquid starter (fermented liquid obtained from a previous kombucha batch) were sourced from China (Zheijiang) and Vietnam (Yen Bai), respectively, and were certified to be organically grown (Da Zhang Shan cooperative, limiting the probability of undesirable molecules in the beverage). The sucrose used in fermentation was a refined white beet sugar from Tirlemont (Belgium). This prevented the formation of an off-flavor and boosted the alcohol content by stimulating the yeast activity throughout the fermentation process [7]. The growing medium used to ferment the tea was a symbiotic culture of bacteria and yeast (SCOBY) from Fairment (Berlin, Germany). This biofilm of cellulose was previously preserved in kombucha. Regarding the major fermentation component analysis, high-purity standards used for the external calibration (namely sucrose, glucose, fructose, ethanol, acetic and lactic acids) and the mobile phase solvent were obtained from Sigma-Aldrich (Darmstadt, Germany). The solvents used in this study were of analytical quality.

### 3.2. Hakko Sobacha Preparation

Sobacha solutions were prepared by infusing kasha at 10 g/L and 50 g/L in mineral water previously heated to 92 °C (Buffalo DM868; LACOR, Bergara, Gipuzkoa, Spain) [37]. After infusing for 20 min, the Sobacha solutions, made in three repetitions, were filtered and sweetened with white beet sugar (60 g/L) [2,37]. Once the solutions were cooled (<35 °C), the inoculum composed of a SCOBY and its liquid starter was added at concentrations of 1% and 10% (*w/w*), respectively. The liquid starter was the result of a previous kombucha fermentation [23]. The containers were covered with a gauze held by an elastic band (aerobic conditions) and then placed in an oven (IPP55plus; F-Nr.: V221.0308; Memmert GmbH+Co.KG, Schwabach, Germany) for the first fermentation (F1) for 10 days at 25 °C [19]. Then, solutions were filtered (200 mesh), and the second fermentation (F2) was set up by transferring them in mechanically sealed bottles (anaerobic conditions leading to natural carbonation) for a further 15 days at 25 °C. Measurements of kinetics were taken on days 0, 1, 2, 3, 6, 7, 8, 9, 10 and 25 of fermentation (referred to as D0, D1, D2, D3, D6, D7, D8, D9, D10 and D25 later in the manuscript). On each day of measurement, a volume of 10 mL was sampled and placed at −80 °C to carry out the different measurements and to evaluate the fermentation kinetics (Section 3.3 and Section 3.4). At the end of F2, Hakko Sobacha beverages were kept in a cold chamber for sensory analysis (Section 3.5).

### 3.3. Dynamic Analysis of the Physico-Chemical Parameters of the Drink

The pH was measured with a pH meter (Hach BeRight; SensION+ pH1; S/N 605069; IP67, Hach Company, Loveland, CO, USA). The pH during fermentation was monitored via direct measurement in the fermentation medium on every sampling day.

At the end of F1, wet weights of nascent SCOBY pellicles were taken after 10 days of incubation. To do so, each nascent pellicle was separated from the original inoculum with sterile forceps, blotted on sterile paper towels over the pellicle 4 times and gently pressed to remove surface water [38]. Nascent pellicles were then dried until dehydration for 4 h at 65 °C (IPP55plus; F-Nr.: V221.0308; Memmert GmbH+Co.KG, Schwabach, Germany). Dry matter was then weighed. The analytical balance used was a Sartorius BC—EB (Sartorius Lab Instruments, Goettingen, Germany).

Quantification of the major components, namely carbohydrates (glucose, fructose, sucrose), ethanol (EtOH), and organic acids (acetic and lactic acids), was performed by high-performance liquid chromatography (HPLC). After sample thawing, centrifugation (Eppendorf MiniSpin, Eppendorf AG, Hamburg, Germany) was conducted with a volume of 2 mL at 10,000 rpm for 10 min at 25 °C [39]. The obtained supernatants were filtered with nylon syringe filters with a pore size of 0.22 µm. Assays were performed on an HPLC system manufactured by Agilent, equipped with an automatic liquid sampler (G1329A) with a thermostat temperature control set at 4 °C. The quaternary pump (G1311A) was used with a flow of 0.6 mL/min. For carbohydrate quantification, an Aminex HPX-87H column with dimensions of 300 mm × 7.8 mm (Bio-Rad Laboratories, Hercules, CA, USA) was used for the separation, with ultrapure H_2_O as the eluent. An Aminex HPX-87C column with dimensions of 300 mm × 7.8 mm (Bio-Rad Laboratories, Hercules, CA, USA) was employed for the separation of ethanol and organic acid kinetics, with 5 mM $H_{2}{\mathrm{SO}}_{4}$ as the eluant. Both columns were equipped with a Micro-Guard Carbo-C Cartridge with dimensions of 30 mm × 4.6 mm (Bio-Rad Laboratories, Hercules, CA, USA). Separations were conducted in isocratic mode, and the thermostatic column compartment (G1316B) was set at 50 °C and 85 °C for Aminex HPX-87H and HPX-87C, respectively. The sample injection volume was 10 µL and the analysis duration lasted 24 min. The detectors used were an MWD (multiple wavelength detector G1365D) set up at 210 nm, followed by an RID (refractive index detector G1362A) fixed at 30 °C. The components were quantified by external standardization.

These assays were performed on all the collected samples in order to obtain the kinetics (production/conversion) of these molecules of interest. Ethanol determination was controlled at the end of the fermentation process to ensure that the levels were below 1.2%, which is the European legislation level (1169/2011) for being considered as a non-alcoholic drink [12].

### 3.4. Chromatographic Analysis of the Development of Volatile Organic Compounds

VOCs were characterized using the SBSE–GC–MS technique, as described by Suffys et al. (2023) with minor modifications [23]. The column used for the separation was a capillary non-polar HP-5MS (5% Phenyl Methyl Polysiloxane) with dimensions 30 m × 250 µm × 0.25 µm.

To optimize compound identification and data processing, the following filters were applied as a first rough compound sorting of the raw data: match factor (percentage of probability of correct identification of the compound under consideration) ≥75% and maximum area under the curve ≥5%. Next, a manually disciplined treatment approach consisted of the validation of all molecules one by one, referring to the mass spectrum by consulting the spectra of reference molecules in the literature. Additionally, the retention index was finally considered to match the validation hypothesis [40].

The semi-quantification of VOCs was calculated relative to the injected internal standard. The determination of OAV (odor-activity value) was calculated as a function of the considered compound concentration in the sample divided by its ortho-nasal detection perception threshold in water, as referred to in the literature [41,42]. This concept allowed for the study of the contribution of the compounds to the aroma of the drink [23,43].

### 3.5. Sensory Analysis

The sensory analyses carried out in this study were the following: triangle discriminative test and appreciation test (hedonic). A questionnaire comprising the initial personal information about the judge, both triangle and hedonic tests, and then questions analyzing his or her knowledge of fermented beverages and consumption habits, ending with questions about preferences for integrating Hakko Sobacha into daily life being proposed. The survey was conducted with a representative sample of 60 evaluators. For this purpose, a recruitment invitation was sent to staff members and students of the Gembloux Agro-Bio Tech faculty (University of Liege, Belgium). The samples tested were the final formulated drinks, corresponding to the last fermentation sampling day (D25), for both buckwheat concentrations considered (10 g/L and 50 g/L). Both beverages (4 cL at 4 °C) were served with water and crackers (mouth rinsing) to allow the untrained jury to taste a panel of products without experiencing any sign of saturation. For representative sampling of the product, a blind presentation of the samples was carried out using a neutral container, with minimum information given about the beverages, and coding with a random 3-digit number. Samples were presented in a homogeneous way and randomized order [44,45].

#### 3.5.1. Triangle Sensory Analysis

The discriminatory triangular test was used to test the hypothesis of identity between two fermented drinks, characterized by their different starting kasha concentrations in the infusion (10 and 50 g/L). The three samples (which had two identical ones) were served (4 cL, 4 °C) simultaneously in a randomized and balanced form [46,47,48,49]. The judge therefore had to determine which of the two samples was different (one of which was presented in duplicate). Values of β = 0.05% and $p_{d}=0.20$ were chosen to ensure that no more than 20% of the population could detect a difference among the samples (within a 95% confidence level). As the results obtained referred to the evaluators’ ability to perceive the difference or otherwise between the two drinks, the data were analyzed by the normal approximation of the binomial test to characterize the judges’ behavior when faced with this test. The critical value table for the triangle test was used to assess the presence of any significant differences [48,49].

#### 3.5.2. Hedonic Evaluation

A non-specific hedonic test was considered in two parts. First, an acceptability measurement was used to evaluate the appreciation of the products on a scale of criteria [50]. Color, taste, acidity, sparkle, roasted hazelnut aroma, and freshness descriptors were evaluated [39,51]. For this purpose, a 5-point appreciation scale (from 1, the lowest preference, to 5, the highest preference) was used. Secondly, a preference measurement was envisaged by a pairwise test, allowing the judge to assign a preferred drink for each descriptor studied. The presentation of the samples (4 cL at 4 °C) was carried out monadically and then simultaneously, respectively.

### 3.6. Raw Data Analysis

All chemical analyses and fermentation medium measurements were performed in three replicates, and the values were expressed as means (*n* = 3) ± standard deviation (SD). Basic statistical data processing was performed using GraphPad Prism (8.0.1 software, GraphPad software Corporation, San Diego, CA, USA) and Microsoft Office Excel (Excel 2021 software, Microsoft Corporation, Redmond, Washington, DC, USA). Multiple student’s t-tests were performed on the pH data, the SCOBY weights and the HPLC data to determine if there were significant differences (*n* = 3; α = 5%) measured between both the buckwheat concentrations and the stages of fermentation modalities. Moreover, this statistical test type was used to identify significant differences (*n* = 60; α = 5%) regarding the hedonic evaluation and pairwise test consumer behaviors.

Principal component analysis (PCA) was performed on all VOC concentrations, considered as individuals. Hakko Sobacha fermentation stages and kasha concentrations were represented as vectors. Considering the sensory analysis, PCAs were performed with evaluators as individuals and samples descriptors as variables. PCA using the Spearman correlation matrix was conducted on the Hakko Sobacha sensory descriptors and kasha concentrations as variables.

The principal components PC1 (Dim1) and PC2 (Dim2) were considered and reported in this article. The variable representation quality was illustrated as cos^2^ values, rated by contribution scale. PCAs were performed in R (R 4.0.2 software, R Development Core Team, Boston, MA, USA).

## 4. Conclusions

To conclude about this survey on the development of a new functional drink based on Sobacha infusion, the main kinetics including pH, SCOBY yields, major fermentation components, VOCs, and aroma-active compounds were analyzed during Hakko Sobacha fermentation. Moreover, the sensory analysis of the formulated beverages reflected the consumer preferences and acceptance of these new refreshing drinks.

Concerning the physico-chemical characterization of kombucha, the pH decrease traduced the acidification of both fermentation media and thus the microorganism’s ability to metabolize diverse products in terms of Sobacha composition. Moreover, the low pH conditions ensured food safety by inhibiting any pathogen development, considering both formulated drinks.

The evolution of the SCOBY weights as a function of the buckwheat concentration pointed out a correlation between the different acidification profiles obtained from the kasha concentration modalities and the carbon sources available for cellulose production during the aerobic fermentation step. However, a study focusing on the comparison of SCOBY production for Sobacha, and tea processing could reveal the performance of the fermentation system under consideration.

The major compounds’ kinetics were analyzed using HPLC, and the main microbial consortium routes were highlighted, such as the glycolysis pathway and AAB pathways. The buckwheat concentration therefore seemed to have an impact on the metabolic activity of sugar conversion by the microorganisms during the first fermentation. Since Sobacha is a food matrix that is rich in starch and made up of glucose units, the main source of fructose in the fermentation medium is sucrose. The availability of additional glucose from the starch hydrolysis during the anaerobic step allows for obtaining a higher final ethanol content—both beverage’s ethanol levels remaining below the 1.2% (*v*/*v*) considered by the European regulation (1169/2011) for characterizing non-alcoholic drinks.

Dynamic changes in VOCs were investigated using SBSE–GC–MS during Hakko Sobacha fermentation. The fermentation media were mainly composed of alcohols, aldehydes, carboxylic acids, esters, ethers, ketones, phenols, pyrazines, and terpenes. Pyrazines present in the infusion were for the most part related to the buckwheat seed roasting process. Thus, their levels were positively correlated with kasha concentration.

Furthermore, the highest concentrated infusion tested seemed to enrich the diversity of the molecules developed during fermentation. The fermentation process dynamically changed the VOC Sobacha profile into a different mixture of compounds. The synthesis of 2-phenylethyl acetate and ethyl caprylate participated in the kombucha-type fermentation signature. The major phenol found in Hakko Sobacha is 4-ethylguaiacol, an aromatic phenol mainly produced by the yeast genus *Brettanomyces*.

The aromatic analysis, based on odor-active values, accounted for about 30 aroma-active compounds. Sobacha presented fat–citrus–green, creamy–waxy, sweet–fruit, and woody–spicy–cumin–oregano aromas. Moreover, typical kasha aromas are correlated with the infusion concentration, bringing flavor such as peanut butter–wood–nut and cocoa. During the fermentation process, the Sobacha sensory profile was modified through MO activity, resulting in the development of diverse aromatic molecules with different perception profiles. The synthesis of 2-phenylethyl acetate, ethyl caprylate and pelargonic acid contributed to the final beverages’ flavors with a pineapple aroma, sweet–waxy–fruity–pineapple notes, and waxy–dairy–cheesy aromas, respectively.

Concerning the sensory analysis results, the PCA variable dispersions regarding both drinks tested seemed to illustrate the consumer’s ability to perceive them differently, as confirmed by the triangle test results. In addition, the descriptor contributions explaining the data variability were the sourness and taste, followed by the hazelnut aroma taste and the freshness of the drinks. Considering the positive correlation between sensorial descriptors, the most linked parameters were acidity intensity–sourness, then taste–roasted hazelnut aroma, and taste–sourness. Moreover, the hedonic evaluation mentioned that the judges’ most frequent comments on the drinks was pleasant. Broadly speaking, as both beverages are appreciated by consumers for their respective descriptor scores, no beverage was rejected from this study. As a result, the major judges surveyed were ready to incorporate Hakko Sobacha into their daily routine as a more natural substitute for soft drinks.

From an industrial perspective, as the fermentation process is affected by many parameters such as temperature, pH, oxygen level, dissolved CO_2_, system precursor supply, shear rate in the fermenter, and the nature and composition of the medium, variations in these factors could affect fermentation speed, performance, organoleptic properties, nutritional quality, and other physico-chemical properties of the product.

Besides all the parameters mentioned above, the kombucha-type fermentation production process might also affect its final properties. Until now, the process has still been rather traditional, and the exact proportion of components may differ according to the product desired.

Nevertheless, to optimize the industrial production of Hakko Sobacha as a functional beverage, a comprehensive study including large-scale production, microbiological identification, and biological testing should be carried out.

As far as prospects are concerned, the characterization of the microbial beverage population could confirm some existing relations between VOC synthesis and the metabolic pathways involved during fermentation, not only by identifying the microorganisms present in the starting SCOBY but also by studying its evolution and potential specification regarding the matrix adaptation.

The SCOBY morphology is also an indicator of the variability of the microbial community. The appearance of the SCOBY could also be studied in greater depth by analyzing its cellulose composition, the formation of sheets, intermolecular links leading to the synthesis of another biopolymer, and the potential capacity for branching cellulose.

As far as the health aspects of the beverage are concerned, it would be interesting to investigate the potential antioxidant and anti-inflammatory properties of Sobacha. Indeed, the evolution of these parameters with fermentation modalities could help to develop optimal functional beverages. Moreover, interactions between the beverage and the intestinal consumer microbiomes should be further studied.

A panel of functional beverages of this type could help in determining the limit of the necessary nutrients for SCOBY development and the dependance factors regarding diverse fermentation parameters. The study of matrix constituent kinetics may allow for the evaluation of this fermentation substrate’s impact on health.

Finally, the economic aspect could be considered in terms of commercializing Hakko Sobacha, as the initial kasha matrix is quite costly. On the one hand, a reduced kasha content could be set. Secondly, as importation from Japan represents a large part of the matrix costs, the development of a local kasha production chain could be envisaged. This would involve studying the parameters for preparing buckwheat and setting up a process for grain roasting, using Japanese know-how to ensure that the matrix is rich in flavor and has beneficial health properties, not to mention its acceptability in non-Asian countries.

## Figures and Tables

**Table 1:** Volatile organic compound contents (ppm) with Hakko Sobacha (kasha concentration 10 g/L) fermentation time (day) conducted at 25 °C.

						Hakko Sobacha Fermentation Stages								
Class	Compound	CAS Number	RI	RI lit	Sobacha	D1	D2	D3	D6	D7	D8	D9	D10	D25
Alcohol	2-ethylhexan-1-ol	104-76-7	1016	1015	nd	nd	nd	14.27 ± 2.68	20.28 ± 4.92	18.80 ± 3.25	16.69 ± 3.19	15.82 ± 0.76	nd	18.22 ± 0.90
	2-phenylethanol	60-12-8	1116	1116	nd	nd	nd	60.92 ± 0.11	nd	67.78 ± 8.49	89.39 ± 8.69	nd	60.80 ± 0.20	nd
	alpha-terpineol	98-55-5	1188	1190	nd	nd	nd	11.73 ± 3.81	13.34 ± 6.77	10.25 ± 0.22	nd	nd	nd	nd
	linalool	78-70-6	1100	1098	nd	19.42 ± 0.20	nd	17.91 ± 0.46	19.27 ± 5.74	16.82 ± 0.36	14.13 ± 1.44	13.28 ± 0.45	11.55 ± 0.20	12.69 ± 2.32
	nonan-1-ol	143-08-8	1169	1169	nd	nd	nd	11.01 ± 8.34	nd	nd	nd	nd	nd	nd
	Total				nd	19.42	nd	115.85	52.89	113.64	120.21	29.10	72.34	30.91
Aldehyde	decanal	112-31-2	1203	1202	58.07	42.77 ± 8.30	nd	22.97 ± 0.92	16.09 ± 7.16	11.95 ± 0.75	53.22 ± 0.56	89.29 ± 1.85	90.95 ± 8.30	42.23 ± 2.71
	dodecanal	112-54-9	1409	1412	nd	12.41 ± 9.00	nd	nd	nd	nd	nd	nd	nd	nd
	nonanal	124-19-6	1102	1102	67.90	39.94 ± 2.43	nd	31.69 ± 2.26	22.19 ± 2.60	16.44 ± 3.75	36.27 ± 7.26	47.13 ± 7.30	43.47 ± 2.43	27.91 ± 4.54
	octanal	124-13-0	1005	1001	nd	nd	nd	nd	nd	nd	21.88 ± 3.73	nd	37.61 ± 0.80	16.84 ± 6.09
	undecanal	112-44-7	1305	1305	nd	13.65 ± 0.80	nd	nd	nd	nd	nd	10.10 ± 6.22	nd	nd
	Total				125.97	108.77	nd	54.66	38.28	28.38	111.37	146.52	172.02	86.97
Carboxylic acid	2-ethylhexanoic acid	149-57-5	1115	1115	nd	nd	nd	11.64 ± 4.94	18.49 ± 3.23	nd	nd	22.40 ± 5.39	nd	nd
	acetic acid	64-19-7	1402	1402	nd	36.14 ± 2.56	nd	nd	nd	nd	nd	36.28 ± 6.35	nd	42.54 ± 2.53
	capric acid	334-48-5	1387	1387	nd	22.78 ± 8.12	60.60 ± 7.79	60.45 ± 2.77	145.32 ± 2.36	100.40 ± 8.12	147.97 ± 4.29	62.11 ± 0.42	nd	116.88 ± 0.12
	caprylic acid	124-07-2	1191	1191	nd	20.47 ± 4.64	29.55 ± 5.72	101.38 ± 2.27	177.36 ± 0.91	168.34 ± 7.88	183.04 ± 9.80	142.89 ± 5.76	115.65 ± 4.64	81.21 ± 6.59
	myristic acid	544-63-8	1769	1769	104.02	21.86 ± 5.82	56.84 ± 2.62	15.80 ± 3.65	nd	12.47 ± 3.00	43.08 ± 8.67	38.44 ± 7.19	39.45 ± 5.82	55.51 ± 1.35
	oleic acid	112-80-1	2102	2101	84.08	nd	22.95 ± 0.41	nd	nd	nd	nd	nd	nd	29.27 ± 0.49
	palmitic acid	57-10-3	1973	1975	650.24	79.42 ± 1.60	53.56 ± 13.75	51.25 ± 7.76	32.75 ± 7.57	51.11 ± 4.54	114.07 ± 2.39	137.46 ± 4.18	139.28 ± 1.60	144.26 ± 5.31
	palmitoleic acid	373-49-9	1953	1953	180.99	nd	66.33 ± 4.72	nd	nd	nd	34.84 ± 4.52	23.83 ± 10.08	24.50 ± 4.62	nd
	pelargonic acid	112-05-0	1271	1272	nd	21.29 ± 9.61	29.86 ± 1.99	55.22 ± 7.30	110.11 ± 9.81	88.29 ± 5.68	104.73 ± 3.76	87.58 ± 3.79	107.95 ± 9.61	75.13 ± 0.12
	pentadecanoic acid	1002-84-2	1869	1869	78.38	11.50 ± 4.81	14.33 ± 5.32	nd	nd	nd	29.52 ± 7.30	23.96 ± 2.06	35.44 ± 4.81	25.35 ± 7.06
	stearic acid	57-11-4	2188	2188	47.20	nd	nd	nd	nd	nd	nd	nd	nd	17.19 ± 4.23
	valeric acid	109-52-4	1744	1744	nd	nd	nd	nd	nd	10.14 ± 1.32	nd	nd	nd	nd
	Total				1144.92	213.46	334.01	295.74	484.03	430.76	657.25	574.94	462.27	587.34
Ester	(2,2,4-trimethyl-3-(2-methylpropanoyloxy)pentyl) 2-methylpropanoate	6846-50-0	1587	1587	nd	36.56 ± 2.46	nd	39.66 ± 17.85	nd	72.92 ± 0.16	82.05 ± 20.16	nd	63.12 ± 22.46	nd
	2-phenylethyl acetate	103-45-7	1256	1256	nd	17.79 ± 8.41	nd	20.17 ± 2.75	25.37 ± 3.52	33.00 ± 13.87	40.23 ± 3.90	46.38 ± 8.61	nd	120.67 ± 8.41
	bis(2-ethylhexyl) hexanedioate	103-23-1	2398	2398	37.64	nd	nd	nd	nd	nd	nd	nd	nd	nd
	hexyl pivalate	5434-57-1	1162	1163	nd	nd	nd	27.64 ± 2.42	nd	nd	nd	nd	nd	nd
	isoamyl laurate	6309-51-9	1844	1844	nd	8.19 ± 1.48	nd	nd	nd	nd	nd	nd	nd	nd
	isopropyl palmitate	142-91-6	1999	1999	73.33	nd	nd	nd	nd	nd	nd	nd	nd	nd
	linalyl acetate	115-95-7	1258	1259	425.75	nd	nd	nd	nd	nd	nd	nd	nd	nd
	octyl caprylate	2306-88-9	1777	1779	nd	nd	nd	nd	nd	nd	nd	nd	nd	23.03 ± 1.02
	triacetin	102-76-1	1344	1344	nd	nd	nd	8.21 ± 2.00	nd	nd	nd	nd	nd	nd
	Total				536.72	62.54	nd	95.69	25.37	105.92	122.28	46.38	63.12	143.70
Ketone	2,6-ditert-butyl-4-hydroxy-4-methylcyclohexa-2,5-dien-1-one	10396-80-2	1477	1478	nd	10.20 ± 1.91	nd	6.72 ± 1.70	nd	nd	nd	nd	nd	nd
	dihydroactinnidiolide	17092-92-1	1525	1525	nd	12.60 ± 5.39	nd	15.65 ± 1.47	19.34 ± 7.71	20.21 ± 1.79	21.50 ± 6.79	13.44 ± 5.25	16.11 ± 5.39	15.64 ± 5.88
	gamma-nonalactone	104-61-0	1362	1360	nd	nd	nd	nd	nd	nd	34.03 ± 0.19	nd	nd	nd
	geranylacetone	3796-70-1	1455	1452	nd	5.37 ± 0.19	nd	11.94 ± 0.51	nd	nd	nd	nd	nd	nd
	Total				nd	28.17	nd	34.31	19.34	20.21	55.53	13.44	16.11	15.64
Phenol	2,4-ditert-butylphenol	96-76-4	1512	1513	72.41	16.08 ± 0.35	nd	16.79 ± 2.43	29.41 ± 7.20	9.93 ± 1.35	nd	13.20 ± 2.05	11.97 ± 0.35	14.34 ± 1.30
	4-ethylguaiacol	2785-87-7	1287	1285	nd	nd	nd	13.83 ± 5.30	18.15 ± 1.16	27.27 ± 7.51	30.19 ± 0.25	20.17 ± 8.97	23.48 ± 9.80	24.38 ± 4.17
	Total				nd	nd	nd	13.83	18.15	27.27	30.19	20.17	23.48	24.38
Pyrazine	2,6-diethylpyrazine	13067-27-1	1081	1081	nd	nd	nd	nd	nd	nd	17.02 ± 2.84	nd	nd	nd
	2-isoamyl-6-methylpyrazine	13925-00-3	915	915	nd	nd	nd	nd	nd	nd	nd	7.96 ± 2.53	nd	nd
	3,5-diethyl-2-methylpyrazine	18138-05-1	1159	1159	nd	nd	nd	7.48 ± 3.27	nd	nd	nd	nd	nd	nd
	Total				nd	nd	nd	7.48	nd	nd	17.02	7.96	nd	nd
Terpene	squalene	111-02-4	2834	2835	62.79	nd	nd	nd	nd	nd	nd	nd	nd	nd
	β-myrcene	535-77-3	1023	1023	510.21	nd	nd	nd	nd	nd	nd	nd	nd	nd
	p-cymene	99-87-6	1025	1025	676.32	42.88 ± 4.14	53.51 ± 8.14	46.98 ± 7.97	34.23 ± 5.19	44.80 ± 8.85	83.22 ± 8.15	75.66 ± 3.63	109.48 ± 4.14	111.84 ± 3.93
	Total				1249.32	42.88	53.51	46.98	34.23	44.80	83.22	75.66	109.48	111.84

Data are expressed as the mean ± SD; “nd”: Not detected. RI: Retention indices on HP-5MS column were determined by n-alkanes. RI lit: Literature retention indices reported for non-polar capillary HP-5MS column.

**Table 2:** Volatile organic compound contents (ppm) with Hakko Sobacha (kasha concentration 50 g/L) fermentation time (day) conducted at 25 °C.

						Hakko Sobacha Fermentation Stages								
Class	Compound	CAS Number	RI	RI lit	Sobacha	D1	D2	D3	D6	D7	D8	D9	D10	D25
Alcohol	2-ethylhexan-1-ol	104-76-7	1016	1015	nd	8.27 ± 1.18	9.19 ± 0.42	11.32 ± 2.26	11.77 ± 0.99	12.97 ± 0.4	18.14 ± 3.16	14.90 ± 6.49	9.69 ± 2.38	19.87 ± 1.22
	2-phenylethanol	60-12-8	1116	1116	nd	nd	nd	nd	nd	nd	nd	nd	117.84 ± 3.84	86.29 ± 3.58
	linalool	78-70-6	1100	1098	nd	21.26 ± 6.64	19.67 ± 1.90	17.59 ± 1.10	13.50 ± 2.3	17.61 ± 7.62	13.48 ± 0.65	nd	10.91 ± 1.03	20.00 ± 5.35
	nerolidol	7212-44-4	1567	1564	nd	nd	nd	nd	nd	nd	nd	nd	nd	20.78 ± 5.93
	Total				nd	29.52	28.87	28.91	25.27	30.58	31.62	14.90	138.44	146.94
Aldehyde	decanal	112-31-2	1203	1202	nd	28.40 ± 9.46	53.40 ± 0.58	26.29 ± 2.70	79.22 ± 1.78	63.00 ± 3.41	49.67 ± 4.89	35.07 ± 5.34	17.83 ± 4.50	45.02 ± 5.05
	nonanal	124-19-6	1102	1102	nd	20.42 ± 0.23	34.11 ± 1.21	23.33 ± 8.83	68.90 ± 8.0	52.91 ± 3.23	50.21 ± 7.86	35.29 ± 4.38	15.34 ± 3.64	27.19 ± 4.73
	octanal	124-13-0	1005	1001	nd	nd	nd	nd	60.69 ± 6.40	31.74 ± 9.32	16.23 ± 5.74	nd	nd	47.52 ± 6.58
	undecanal	112-44-7	1305	1305	nd	nd	nd	nd	16.90 ± 0.51	13.23 ± 4.79	13.05 ± 2.43	nd	nd	nd
	Total				nd	48.81	87.51	49.63	225.70	160.87	129.17	70.36	33.17	119.73
Benzothiazole	1,3-benzothiazole	95-16-9	1227	1224	nd	nd	nd	nd	nd	nd	nd	5.68 ± 2.56	nd	nd
	Total				nd	nd	nd	nd	nd	nd	nd	5.68	nd	nd
Carboxylic acid	acetic acid	64-19-7	1402	1402	nd	44.55 ± 3.85	18.55 ± 2.14	23.12 ± 6.27	nd	41.86 ± 3.87	38.49 ± 6.69	nd	10.51 ± 3.34	44.09 ± 1.91
	capric acid	334-48-5	1387	1387	nd	67.21 ± 2.07	78.79 ± 7.33	48.58 ± 7.89	57.52 ± 4.19	nd	nd	nd	nd	nd
	caprylic acid	124-07-2	1191	1191	nd	nd	nd	nd	nd	nd	58.35 ± 8.37	nd	nd	67.41 ± 9.18
	myristic acid	544-63-8	1769	1769	65.62	21.67 ± 3.00	27.27 ± 2.53	26.61 ± 4.60	nd	nd	nd	nd	19.57 ± 4.75	44.89 ± 2.73
	oleic acid	112-80-1	2102	2101	44.98	nd	nd	nd	nd	nd	nd	nd	nd	nd
	palmitic acid	57-10-3	1973	1975	304.18	54.14 ± 8.37	69.62 ± 4.05	78.80 ± 0.45	23.65 ± 4.01	59.47 ± 3.70	32.54 ± 0.67	30.09 ± 3.39	61.46 ± 8.72	119.47 ± 4.46
	palmitoleic acid	373-49-9	1953	1953	144.70	nd	nd	nd	nd	nd	nd	nd	nd	22.97 ± 4.02
	pelargonic acid	112-05-0	1271	1272	nd	54.86 ± 6.66	71.38 ± 6.19	58.74 ± 5.98	47.52 ± 6.73	72.43 ± 1.72	nd	nd	nd	nd
	pentadecanoic acid	1002-84-2	1869	1869	50.19	9.28 ± 2.64	nd	13.18 ± 8.18	nd	nd	nd	nd	11.09 ± 2.32	23.63 ± 5.48
	stearic acid	57-11-4	2188	2188	32.16	nd	nd	nd	nd	nd	nd	nd	nd	nd
	Total				641.83	251.72	265.60	249.04	128.70	173.76	129.37	30.09	102.64	322.45
Ester	(2-decanoyloxy-3-octanoyloxypropyl) decanoate	82426-88-8	3348	3348	nd	6.79 ± 0.78	nd	7.78 ± 0.19	nd	nd	nd	nd	nd	nd
	[2,2,4-trimethyl-3-(2-methylpropanoyloxy)pentyl] 2-methylpropanoate	6846-50-0	1587	1587	nd	30.84 ± 1.52	36.09 ± 2.72	50.89 ± 6.86	93.09 ± 2.01	80.65 ± 0.74	93.85 ± 2.60	84.85 ± 6.78	50.19 ± 2.21	78.92 ± 0.65
	1,3-di(octanoyloxy)propan-2-yl decanoate	33368-87-5	3137	3137	nd	24.43 ± 4.83	34.95 ± 2.27	25.74 ± 4.79	nd	nd	nd	nd	nd	40.61 ± 8.29
	2-phenylethyl acetate	103-45-7	1256	1256	nd	14.24 ± 4.31	16.72 ± 3.25	77.44 ± 8.65	nd	nd	nd	nd	22.58 ± 5.83	57.55 ± 2.20
	ethyl 2-phenylacetate	101-97-3	1244	1244	nd	nd	nd	nd	nd	nd	nd	nd	nd	10.25 ± 3.45
	ethyl caprylate	106-32-1	1196	1196	nd	nd	nd	nd	33.19 ± 5.69	54.40 ± 6.93	167.58 ± 8.85	94.33 ± 3.06	43.93 ± 8.48	95.52 ± 1.29
	isoamyl laurate	6309-51-9	1844	1844	nd	26.73 ± 5.38	33.72 ± 1.53	32.48 ± 1.83	nd	nd	nd	nd	nd	31.54 ± 8.18
	methyl caproate	106-70-7	924	924	119.45	nd	nd	nd	nd	nd	nd	nd	nd	nd
	octan-2-yl hexadecanoate	55194-81-5	2501	2501	nd	13.14 ± 2.19	17.60 ± 0.84	14.91 ± 3.36	nd	nd	nd	nd	nd	20.94 ± 3.97
	triacetin	102-76-1	1344	1344	nd	9.10 ± 1.61	9.27 ± 0.63	9.49 ± 1.11	nd	nd	nd	nd	nd	nd
	tricaprylin	538-23-8	2858	2859	nd	17.36 ± 4.71	25.28 ± 7.75	18.15 ± 2.89	nd	nd	nd	nd	nd	nd
	Total				119.45	142.63	173.63	236.89	126.28	135.06	261.43	179.18	116.70	335.34
Ketone	2,6-ditert-butyl-4-hydroxy-4-methylcyclohexa-2,5-dien-1-one	10396-80-2	1477	1478	nd	9.06 ± 2.72	12.09 ± 1.60	7.30 ± 1.43	nd	12.82 ± 2.75	16.61 ± 1.08	11.60 ± 7.57	7.18 ± 1.67	15.69 ± 5.78
	dihydroactinnidiolide	17092-92-1	1525	1525	nd	10.71 ± 3.04	10.93 ± 0.15	11.12 ± 1.21	22.28 ± 9.41	20.00 ± 2.39	24.24 ± 4.97	17.79 ± 8.18	12.23 ± 3.21	12.47 ± 2.54
	gamma-nonalactone	104-61-0	1362	1360	nd	nd	nd	nd	nd	25.93 ± 5.62	22.55 ± 3.87	nd	nd	nd
	gamma-undecalactone	104-67-6	1573	1573	nd	27.81 ± 0.31	35.72 ± 8.00	33.90 ± 3.55	nd	nd	nd	nd	nd	nd
	3,5-dihydroxy-6-methyl-2,3-dihydropyran-4-one	28564-83-2	1154	1154	nd	nd	nd	nd	nd	20.38 ± 9.86	68.11 ± 7.99	127.31 ± 7.95	nd	nd
	Total				nd	47.58	58.74	52.32	22.28	79.12	131.52	156.70	19.40	28.16
Phenol	2,4-ditert-butylphenol	96-76-4	1512	1513	nd	8.74 ± 2.76	nd	7.04 ± 0.98	19.62 ± 6.12	nd	nd	10.84 ± 5.99	7.47 ± 1.58	nd
	4-ethylguaiacol	2785-87-7	1287	1285	nd	14.13 ± 5.91	18.31 ± 8.59	33.73 ± 5.84	31.81 ± 5.07	68.07 ± 0.13	122.98 ± 0.62	141.96 ± 8.29	63.55 ± 9.73	116.18 ± 3.33
	dihydroeugenol	2785-89-9	1261	1260	nd	nd	nd	nd	nd	nd	nd	nd	35.09 ± 3.67	12.39 ± 0.25
	isochavibetol	19784-98-6	1312	1312	nd	10.19 ± 0.93	nd	13.79 ± 2.19	nd	nd	nd	nd	10.32 ± 2.00	nd
	Total				nd	33.06	18.31	54.55	51.43	68.07	122.98	152.80	116.44	128.56
Pyrazine	2,5-dimethyl-3-propylpyrazine	18433-97-1	1162	1162	nd	15.43 ± 4.90	125.65 ± 7.86	9.00 ± 1.57	nd	nd	nd	nd	nd	nd
	2,6-diethylpyrazine	13067-27-1	1081	1081	nd	nd	nd	63.59 ± 1.39	68.41 ± 7.27	nd	32.98 ± 1.22	nd	23.98 ± 5.70	16.76 ± 7.98
	2-ethyl-5-methylpyrazine	13360-64-0	1001	1000	nd	66.98 ± 7.88	nd	nd	nd	nd	nd	nd	23.41 ± 9.21	nd
	2-ethylpyrazine	13360-65-1	1077	1078	129.11	nd	nd	35.45 ± 5.61	nd	nd	nd	nd	14.85 ± 3.17	nd
	2-isoamyl-6-methylpyrazine	13925-00-3	915	915	64.64	27.91 ± 8.79	31.81 ± 6.60	25.66 ± 7.56	nd	nd	nd	nd	16.90 ± 0.11	23.85 ± 6.77
	2-isobutyl-3-methylpyrazine	13925-06-9	1114	1114	nd	9.50 ± 1.10	18.06 ± 2.11	8.33 ± 1.28	nd	nd	nd	nd	nd	nd
	5-ethyl-2,3-dimethylpyrazine	15707-34-3	1104	1105	nd	nd	9.89 ± 0.59	10.03 ± 0.02	nd	nd	nd	nd	nd	nd
	2-methylpyrazine	109-08-0	1151	1150	41.55	nd	nd	nd	nd	nd	nd	nd	nd	nd
	3,5-diethyl-2-methylpyrazine	18138-05-1	1159	1159	72.24	22.13 ± 8.12	21.66 ± 2.82	16.06 ± 6.01	10.43 ± 0.62	nd	nd	nd	6.40 ± 0.97	nd
	2-methoxy-5-methylpyrazine	2882-22-6	971	971	342.21	62.80 ± 2.30	nd	nd	nd	nd	nd	nd	nd	nd
	Total				649.76	174.11	197.76	152.95	75.37	nd	32.98	nd	65.02	35.02
Pyrrole	1-(furan-2-ylmethyl)pyrrole	1438-94-4	1185	1185	43.78	nd	nd	nd	nd	nd	nd	nd	nd	nd
	Total				43.78	nd	nd	nd	nd	nd	nd	nd	nd	nd
Terpene	squelene	111-02-4	2834	2835	53.88	nd	nd	nd	nd	nd	nd	nd	nd	nd
	β-myrcene	535-77-3	1023	1023	439.92	nd	nd	nd	nd	nd	nd	nd	nd	nd
	D-limonene	5989-27-5	1036	1035	32.43	nd	nd	nd	nd	nd	nd	nd	nd	nd
	p-cymene	99-87-6	1025	1025	635.14	44.66 ± 8.94	49.16 ± 9.68	51.50 ± 7.49	10.72 ± 2.29	nd	129.58 ± 5.24	133.63 ± 9.15	50.63 ± 9.19	117.51 ± 2.37
	Total				1161.38	44.66	49.16	51.50	10.72	nd	129.58	133.63	50.63	117.51

Data are expressed as the mean ± SD; “nd”: Not detected. RI: Retention indices on HP-5MS column were determined by n-alkanes. RI lit: Literature retention indices reported for non-polar capillary HP-5MS column.

**Table 3:** OAV evolution of most representative volatile organic compounds (% area > 5%) with Hakko Sobacha (kasha concentration 10 g/L) fermentation time (day) conducted at 25 °C (OAV = concentration/perception threshold).

									Hakko Sobacha Fermentation Stages								
Class	Compound		CAS Number	RI	RI lit	Perception Threshold (ppm)	Aroma Description	Sobacha	D1	D2	D3	D6	D7	D8	D9	D10	D25
Alcohol	2-phenylethanol	A1	60-12-8	1116	1116	20	Sweet, floral, bready, honey	nd	nd	nd	3.05	nd	3.39	4.47	nd	3.04	nd
	alpha-terpineol	A2	98-55-5	1188	1190	22.5	Anise, mint	nd	nd	nd	0.52	0.59	0.46	nd	nd	nd	nd
	linalool	A3	78-70-6	1100	1098	10	Citrus, floral, orange, terpene	nd	1.94	nd	1.79	1.93	1.68	1.41	1.33	1.15	1.27
Aldehyde	decanal	B1	112-31-2	1203	1202	30	Soap, orange peel, tallow	1.94	1.43	nd	0.77	0.54	0.40	1.77	2.98	3.03	1.41
	dodecanal	B2	112-54-9	1409	1412	2	Oil, pungent, sweet	nd	6.21	nd	nd	nd	nd	nd	nd	nd	nd
	nonanal	B3	124-19-6	1102	1102	8	Fat, citrus, green	8.49	4.99	nd	3.96	2.77	2.05	4.53	5.89	5.43	3.49
	octanal	B4	124-13-0	1005	1001	25	Fat, soap, lemon, green	nd	nd	nd	nd	nd	nd	0.88	nd	1.50	0.67
	undecanal	B5	112-44-7	1305	1305	12.5	Oil, tallow	nd	1.09	nd	nd	nd	nd	nd	0.81	nd	nd
Carboxylic acid	myristic acid	C1	544-63-8	1769	1769	30	Faint, waxy, fatty	3.47	0.73	5.23	0.53	nd	0.42	1.44	1.28	1.31	1.85
	oleic acid	C2	112-80-1	2102	2101	15	Fat	5.61	nd	8.20	nd	nd	nd	nd	nd	nd	1.95
	palmitic acid	C3	57-10-3	1973	1975	75	Creamy, waxy	8.67	1.06	7.38	0.68	0.44	0.68	1.52	1.83	1.86	1.92
	pelargonic acid	C4	112-05-0	1271	1272	10	Waxy, dairy, cheesy	nd	2.13	12.99	5.52	11.01	8.83	10.47	8.76	10.79	7.51
	valeric acid	C5	109-52-4	1744	1744	100	Sweet, acid, rancid	nd	nd	nd	nd	nd	0.10	nd	nd	nd	nd
Ester	2-phenylethyl acetate	D1	103-45-7	1256	1256	7.5	Pineapple	nd	2.37	nd	2.69	3.38	4.40	5.36	6.18	nd	16.09
	isoamyl laurate	D2	6309-51-9	1844	1844	10	Leaf	nd	0.82	nd	nd	nd	nd	nd	nd	nd	nd
	linalyl acetate	D3	115-95-7	1258	1259	5	Sweet, fruit	85.15	nd	nd	nd	nd	nd	nd	nd	nd	nd
Ketone	gamma-nonalactone	E1	104-61-0	1362	1360	10	Coconut, peach	nd	nd	nd	nd	nd	nd	3.40	nd	nd	nd
	geranylacetone	E2	3796-70-1	1455	1452	12	Rose	nd	0.45	nd	0.99	nd	nd	nd	nd	nd	nd
Phenol	4-ethylguaiacol	F1	2785-87-7	1287	1285	30	Spicy, sweet vanilla, woody	nd	nd	nd	0.46	0.60	0.91	1.01	0.67	0.78	0.81
Pyrazine	2-isoamyl-6-methylpyrazine	G1	13925-00-3	915	915	10	Popcorn	nd	nd	nd	nd	nd	nd	nd	0.80	nd	nd
	3,5-diethyl-2-methylpyrazine	G2	18138-05-1	1159	1159	1	Nut, peanut butter, cocoa	nd	nd	nd	7.48	nd	nd	nd	nd	nd	nd
Terpene	beta-myrcene	H1	123-35-3	990	990	10	Balsamic, must, spice	3.75	nd	nd	nd	nd	nd	nd	nd	nd	nd
	β-myrcene	H2	535-77-3	1023	1023	52.5	Citrus	9.72	nd	nd	nd	nd	nd	nd	nd	nd	nd
	p-cymene	H3	99-87-6	1025	1025	5.5	Woody, spicy, cumin, oregano	122.97	7.80	137.00	8.54	6.22	8.15	15.13	13.76	19.90	20.33

RI: Retention indices on HP-5MS column were determined by n-alkanes. RI lit: Literature retention indices reported for non-polar capillary HP-5MS column.

**Table 4:** OAV evolution of most representative volatile organic compounds (% area > 5%) with Hakko Sobacha (kasha concentration 50 g/L) fermentation time (day) conducted at 25 °C (OAV = concentration/perception threshold).

									Hakko Sobacha Fermentation Stages								
Class	Compound		CAS Number	RI	RI lit	Perception Threshold (ppm)	Aroma Description	Sobacha	D1	D2	D3	D6	D7	D8	D9	D10	D25
Alcohol	2-phenylethanol	a1	60-12-8	1116	1116	50	Sweet, floral, bready, honey	nd	nd	nd	nd	nd	nd	nd	nd	2.36	1.73
	linalool	a2	78-70-6	1100	1098	10	Citrus, floral, orange, terpene	nd	2.13	1.97	1.76	1.35	1.76	1.35	nd	1.09	2.00
	nerolidol	a3	7212-44-4	1567	1564	25	Wood, flower, wax	nd	nd	nd	nd	nd	nd	nd	nd	nd	0.83
Aldehyde	decanal	b1	112-31-2	1203	1202	30	Soap, orange peel, tallow	nd	0.95	1.78	0.88	2.64	2.10	1.66	1.17	0.59	1.50
	nonanal	b2	124-19-6	1102	1102	8	Fat, citrus, green	nd	2.55	4.26	2.92	8.61	6.61	6.28	4.41	1.92	3.40
	octanal	b3	124-13-0	1005	1001	25	Fat, soap, lemon, green	nd	nd	nd	nd	2.43	1.27	0.65	nd	nd	1.90
	undecanal	b4	112-44-7	1305	1305	12.5	Oil, tallow	nd	nd	nd	nd	1.35	1.06	1.04	nd	nd	nd
Benzothiazole	1,3-benzothiazole	c1	95-16-9	1227	1224	3	Gasoline, rubber	nd	nd	nd	nd	nd	nd	nd	1.89	nd	nd
Carboxylic acid	acetic acid	d1	64-19-7	1402	1402	50.5	Sour vinegar	nd	0.88	0.37	0.46	nd	0.83	0.76	nd	0.21	0.87
	myristic acid	d2	544-63-8	1769	1769	30	Faint, waxy, fatty	2.19	0.72	0.91	0.89	nd	nd	nd	nd	0.65	1.50
	oleic acid	d3	112-80-1	2102	2101	15	Fat	3.00	nd	nd	nd	nd	nd	nd	nd	nd	nd
	palmitic acid	d4	57-10-3	1973	1975	75	Creamy, waxy	4.06	0.72	0.93	1.05	0.32	0.79	0.43	0.40	0.82	1.59
	pelargonic acid	d5	112-05-0	1271	1272	10	Waxy, dairy, cheesy	nd	5.49	7.14	5.87	4.75	7.24	nd	nd	nd	nd
Ester	2-phenylethyl acetate	e1	103-45-7	1256	1256	7.5	Pineapple	nd	1.90	2.23	10.33	nd	nd	nd	nd	3.01	7.67
	ethyl 2-phenylacetate	e2	101-97-3	1244	1244	7.5	Fruit, herb	nd	nd	nd	nd	nd	nd	nd	nd	nd	1.37
	ethyl caprylate	e3	106-32-1	1196	1196	7.5	Sweet, waxy, fruity, pineapple	nd	nd	nd	nd	4.43	7.25	22.34	12.58	5.86	12.74
	isoamyl laurate	e4	6309-51-9	1844	1844	10	Leaf	nd	2.67	3.37	3.25	nd	nd	nd	nd	nd	3.15
Ketone	gamma-nonalactone	f1	104-61-0	1362	1360	10	Coconut, peach	nd	nd	nd	nd	nd	2.59	2.26	nd	nd	nd
	gamma-undecalactone	f2	104-67-6	1573	1573	30	Peach	nd	0.93	1.19	1.13	nd	nd	nd	nd	nd	nd
Phenol	4-ethylguaiacol	g1	2785-87-7	1287	1285	30	Spicy, sweet vanilla, woody	nd	0.47	0.61	1.12	1.06	2.27	4.10	4.73	2.12	3.87
Pyrazine	2,5-dimethyl-3-propylpyrazine	h1	18433-97-1	1162	1162	1	Roasted nut, cocoa	nd	15.43	125.65	9.00	nd	nd	nd	nd	nd	nd
	2-ethyl-5-methylpyrazine	h2	13360-64-0	1001	1000	1	Roast, sweat	nd	66.98	nd	nd	nd	nd	nd	nd	23.41	nd
	2-ethylpyrazine	h3	13360-65-1	1077	1078	10	Peanut butter, wood	12.91	nd	nd	3.54	nd	nd	nd	nd	1.49	nd
	2-isoamyl-6-methylpyrazine	h4	13925-00-3	915	915	10	Popcorn	6.46	2.79	3.18	2.57	nd	nd	nd	nd	1.69	2.38
	2-isobutyl-3-methylpyrazine	h5	13925-06-9	1114	1114	1	Burnt, popcorn	nd	9.50	18.06	8.33	nd	nd	nd	nd	nd	nd
	2-methylpyrazine	h6	109-08-0	1151	1150	75	Baked, potato	0.55	nd	nd	nd	nd	nd	nd	nd	nd	nd
	3,5-diethyl-2-methylpyrazine	h7	18138-05-1	1159	1159	1	Nut, peanut butter, cocoa	72.24	22.13	21.66	16.06	10.43	nd	nd	nd	6.40	nd
Pyrrole	1-(furan-2-ylmethyl)pyrrole	i1	1438-94-4	1185	1185	5	Vegetable	8.76	nd	nd	nd	nd	nd	nd	nd	nd	nd
Terpene	β-myrcene	j1	535-77-3	1023	1023	52.5	Citrus	8.38	nd	nd	nd	nd	nd	nd	nd	nd	nd
	D-limonene	j2	5989-27-5	1036	1035	30	Citrus, orange, fresh, sweet	1.08	nd	nd	nd	nd	nd	nd	nd	nd	nd
	p-cymene	j3	99-87-6	1025	1025	5.5	Woody, spicy, cumin, oregano	115.48	8.12	8.94	9.36	1.95	nd	23.56	24.30	9.20	21.36

RI: Retention indices on HP-5MS column were determined by n-alkanes. RI lit: Literature Retention indices reported for non-polar capillary HP-5MS column.

## Data Availability

The datasets generated for this study are available on request to the corresponding author.

## Sample Availability

Samples of the compounds are not available from the authors.
